# Absence of calcium‐independent phospholipase A_2_*β* impairs platelet‐activating factor production and inflammatory cell recruitment in *Trypanosoma cruzi*‐infected endothelial cells

**DOI:** 10.1002/phy2.196

**Published:** 2014-01-06

**Authors:** Janhavi Sharma, Christopher S. Eickhoff, Daniel F. Hoft, John O. Marentette, John Turk, Jane McHowat

**Affiliations:** 1 Department of Pathology, Saint Louis University School of Medicine, 1402 S. Grand BlvdSt Louis, 63104, Missouri; 2 Division of Infectious Diseases, Department of Internal Medicine, Saint Louis University School of Medicine, 1402 S. Grand Blvd., St Louis, 63104, Missouri; 3 Division of Endocrinology, Metabolism and Lipid Research, Department of Medicine, Washington University School of Medicine, Washington University in St. Louis, St. Louis, 63110, Missouri

**Keywords:** endothelial, Inflammation, phospholipase A_2_, platelet‐activating factor

## Abstract

Both acute and chronic phases of *Trypanosoma cruzi* (*T. cruzi*) infection are characterized by tissue inflammation, mainly in the heart. A key step in the inflammatory process is the transmigration of inflammatory cells across the endothelium to underlying infected tissues. We observed increased arachidonic acid release and platelet‐activating factor (PAF) production in human coronary artery endothelial cells (HCAEC) at up to 96 h of *T. cruzi* infection. Arachidonic acid release is mediated by activation of the calcium‐independent phospholipase A_2_ (iPLA_2_) isoforms iPLA_2_*β* and iPLA_2_*γ*, whereas PAF production was dependent upon iPLA_2_*β* activation alone. *Trypanosoma cruzi* infection also resulted in increased cell surface expression of adhesion molecules. Increased adherence of inflammatory cells to *T. cruzi*‐infected endothelium was blocked by inhibition of endothelial cell iPLA_2_*β* or by blocking the PAF receptor on inflammatory cells. This suggests that PAF, in combination with adhesion molecules, might contribute to parasite clearing in the heart by recruiting inflammatory cells to the endothelium.

## Abstract (precis)

Both acute and chronic phases of *Trypanosoma cruzi* (*T. cruzi*) infection are characterized by tissue inflammation that requires transmigration of inflammatory cells across the endothelium to underlying infected tissues. We observed increased platelet‐activating factor (PAF) adherence of inflammatory cells to *T. cruzi*‐infected endothelium that was blocked by inhibition of endothelial cell calcium‐independent phospholipase A_2_*β* or by blocking the PAF receptor on inflammatory cells. This suggests that PAF, in combination with adhesion molecules, might contribute to parasite clearing in the heart by recruiting inflammatory cells to the endothelium.

## Introduction

*Trypanosoma cruzi* is the protozoan parasite responsible for Chagas’ disease, which is associated with significant cardiac pathology. Over 10 million people worldwide are thought to be currently infected with *T. cruzi,* and about 300,000 infected individuals live in the United States (Bern and Montgomery 2009). The recent spread of the disease to several nonendemic countries is attributable to immigration from endemic areas, immunosuppression in the setting of organ transplantation, and blood transfusions from infected individuals. The acute phase of Chagas’ disease may not cause symptoms, but in the chronic phase cardiac involvement occurs in 20–30% of infected individuals and may result in congestive heart failure, cardiac arrhythmias, and death (Rassi et al. 2000; Bern 2011). A long asymptomatic period separating acute and chronic phases is designated the indeterminate phase and may persist for decades.

Interactions between the host and pathogen during acute infection may determine the outcome of chronic Chagas’ disease (Marinho et al. 1999). Parasite persistence reflected by the presence of *T. cruzi* antigens and DNA in the heart have been found to correlate with the intensity of chronic disease (Jones et al. 1993; Benvenuti et al. 2008), and it is therefore necessary to understand parasite–host interactions in the acute phase of Chagas’ disease. A key pathological feature of *T. cruzi* infection is the intense cardiac inflammation in both acute and chronic stages. As a consequence of acute stage parasitemia, trypomastigotes migrate across endothelial barriers to infect underlying tissues, resulting in increased expression of vascular adhesion molecules and pro‐inflammatory cytokines when *T. cruzi* infects endothelial cells (Huang et al. 1999; Michailowsky et al. 2004). Infection of the endothelium has a well‐established role in the pathogenesis of Chagas’ disease and contributes to increased platelet aggregation and thrombus formation (Rossi et al. 1984; Tanowitz et al. 1990).

Platelet‐activating factor (PAF) is an important membrane phospholipid‐derived inflammatory mediator expressed on the surface of endothelial cells, where it plays an important role in the recruitment, activation, and transmigration of leukocytes to sites of infection (Prescott et al. 2002). PAF is an acetylated alkyl ether glycerophosphocholine lipid species whose immediate precursor is produced by the action of phospholipase A_2_ (PLA_2_) enzyme(s), and PAF can elicit biological responses at concentrations as low as 10^−12^ mol/L (Montrucchio et al. 2000). The PLA_2_ family comprises enzymes that hydrolyze phospholipids at the *sn‐2* position to yield a free fatty acid and a 2‐lysophospholipid. Lysophospholipid species of the structure 1‐O‐alkyl, 2‐lyso‐glycerophosphocholine (GPC) are designated lyso‐PAF and when acetylated in the *sn*‐2 position yield PAF (McHowat et al. 2001). When endothelial cell PAF interacts with the PAF receptor expressed on the surface of leukocytes (Shimizu et al. 1992), leukocytes become tethered to the endothelium and activated leukocytes can transmigrate into underlying tissues (Prescott et al. 2001).

We have shown that the major PLA_2_ in cardiac endothelial cells is membrane associated, calcium‐independent (iPLA_2_), and specific for arachidonylated phospholipids (Creer and McHowat 1998). Two iPLA_2_ isoforms (iPLA_2_*β* and iPLA_2_*γ*) are expressed by cardiac endothelial cells (Sharma et al. 2011) and can be distinguished pharmacologically. The (*R*) enantiomer of the compound BEL (bromoenol lactone) preferentially inhibits iPLA_2_*γ*, and (*S*)‐BEL preferentially iPLA_2_*β* (Jenkins et al. 2002). In vitro studies using (*R*)‐ and (*S*)‐BEL show that iPLA_2_*β* activation results in PAF production, which is required for neutrophil adherence to cardiac endothelium (White and McHowat 2007; Sharma et al. 2011). Activated cardiac endothelial cells from wild‐type and iPLA_2_*γ* knockout mice produce PAF, but such cells from iPLA_2_*β* knockout mice fail to do so (Sharma et al. 2011). This suggests that iPLA_2_*β* may play an important role in recruiting inflammatory cells to the myocardium by enabling PAF production. Although downstream mediators generated from products of iPLA_2_ action have been studied in Chagas’ disease, there has been no examination of the contribution of individual iPLA_2_ isoforms to these processes. We have therefore examined the contribution of endothelial cell iPLA_2_*β* to inflammatory cell recruitment following *T. cruzi* infection.

## Materials and Methods

### Human coronary artery endothelial cells

Human coronary artery endothelial cells (HCAEC) were obtained from Lonza Walkersville, Inc. (Walkersville, MD). Cells were grown to confluence in EGM‐2MV media obtained from Lonza (Walkersville, MD), with 5% fetal bovine serum (FBS). Cells were allowed to grow to confluence achieving a contact‐inhibited monolayer of flattened, closely apposed endothelial cells in 4–5 days. After achieving confluence, cells were passaged in a 1:3 dilution and cells from passages 3–4 were used for experiments.

### Mouse endothelial cell isolation

Animal protocols were in strict accordance with the National Institutes of Health guidelines for humane treatment of animals and were reviewed and approved by the Animal Care and Use Committee of Saint Louis University. Endothelial cells were isolated from mouse heart by collagenase digestion. The diced heart muscle was incubated in 2 mg/mL collagenase for 1 h at 37°C and the digested tissue was passed through a cell strainer. Cells were incubated with murine immunoglobulins to block Fc receptors and then incubated with anti‐mouse platelet endothelial cell adhesion molecule‐1 (PECAM‐1) coupled to magnetic beads. Cells obtained were cultured until they reached confluence and sorted again using intercellular adhesion molecule‐2 (ICAM‐2) antibodies coupled with magnetic beads. The eluted cells were washed, resuspended in cell culture medium, and plated in culture. Nonadherent cells were removed the next day and cells were grown to confluence and passaged at a 1–3 dilution.

### Parasitology

Tissue culture trypomastigotes (TCT) from the Brazil strain of *T. cruzi* were propagated in 3T3 mouse embryonic fibroblasts grown in Dulbecco's modified Eagle medium (DMEM) supplemented with 2% neonatal calf serum (Eickhoff et al. 2010). 3T3 cells were infected with *T. cruzi* when 60% confluence was reached. Infected cells ruptured following parasite multiplication, releasing an abundant number of parasites. The supernatant containing the parasites was collected, and parasite numbers determined using a Neubauer hemocytometer (Patterson Veterinary, Devens, MA). Cardiac endothelial cells, grown to confluence in the appropriate culture dish were counted and a multiplicity of infection (MOI) of 0.2 was used to infect cells. In selected experiments, parasites were killed by heating at 80°C for 10 min.

### Phospholipase A_2_ activity

Endothelial cells were suspended in 1 mL buffer containing (mmol/L): Sucrose 250, KCL 10, imidazole 10, ethylenediaminetetraacetic acid (EDTA) 5, dithiothreitol (DTT) 2 with 10% glycerol, pH 7.8 (PLA_2_ activity buffer). The suspension was sonicated on ice six times for 10 sec (using microtip probe at 20% power output, 500 Sonic Dismembrator; Fisher Scientific, Pittsburgh, PA) and the sonicate centrifuged at 20,000*g* for 20 min to remove cellular debris and nuclei. The pellet was resuspended in activity buffer. PLA_2_ activity was assessed by incubating enzyme (50 *μ*g protein) with 100 *μ*mol/L (16:0, [^3^H]18:1) plasmenylcholine substrate in assay buffer containing (mmol/L): Tris 10, ethylene glycol tetraacetic acid (EGTA) 4, 10% glycerol, pH 7.0 at 37°C for 5 min in a total volume of 200 *μ*L. The radiolabeled phospholipid substrate was introduced into the incubation mixture by injection in 5 *μ*L ethanol to initiate the assay. Reactions were terminated by the addition of 100‐*μ*L butanol and released radiolabeled [^3^H]oleic acid was isolated by the application of 25 *μ*L of the butanol phase to channeled Silica Gel G plates, development in the petroleum ether/diethyl ether/acetic acid (70/30/1, v/v) and subsequent quantification by liquid scintillation spectrometry. Protein content of each sample was determined by the Lowry method utilizing freeze‐dried bovine serum albumin as the protein standard.

### Measurement of total arachidonic acid release

Endothelial cells were incubated at 37°C with 3 *μ*Ci [^3^H] arachidonic acid for 18 h. This incubation resulted in >70% incorporation of radioactivity into membrane phospholipids. Cells were fed with fresh medium containing [^3^H] arachidonic acid after 48 h where necessary. After incubation, endothelial cells were washed three times with Tyrode solution containing 0.36% bovine serum albumin to remove unincorporated [^3^H] arachidonic acid. Endothelial cells were incubated at 37°C for 15 min before being subjected to experimental conditions. At the end of the stimulation period the supernatant was removed. Endothelial cells were lysed in 10% sodium dodecyl sulfate, and radioactivity in both supernatant and pellet was quantified by liquid scintillation spectrometry.

### PAF assay

Endothelial cells grown in 12‐well culture dishes were washed twice with Hanks’ balanced salts solution containing NaCl 135 mmol/L, MgSO_4_ 0.8 mmol/L, HEPES (pH = 7.4) 10 mmol/L, CaCl_2_ 1.2 mmol/L, KCl 5.4 mmol/L, KH_2_PO_4_ 0.4 mmol/L, Na_2_HPO_4_ 0.3 mmol/L, and glucose 6.6 mmol/L and incubated with 50 *μ*Ci [^3^H] acetic acid for 20 min. After the selected time interval for incubation with the appropriate agents, lipids were be extracted from the cells by the method of Bligh and Dyer (1959). The chloroform layer was concentrated by evaporation under N_2_, applied to a silica gel 60 thin layer chromatography (TLC) plate, and developed in chloroform/methanol/acetic acid/water (50/25/8/4 vol/vol). The region corresponding to PAF was scraped and radioactivity quantified using liquid scintillation spectrometry. Loss of PAF during extraction and chromatography was corrected for by adding a known amount of [^14^C] PAF as an internal standard. [^14^C] PAF was synthesized by acetylating the *sn*‐2 position of lyso‐PAF with [^14^C] acetic anhydride using 0.33 mol/L dimethylaminopyridine as a catalyst. The synthesized [^14^C] PAF was purified by high‐performance liquid chromatography (HPLC).

### Inflammatory cell adherence

RAW 264.7 cells were grown to confluence in DMEM with 10% FBS. Cell suspensions (10 × 10^6^/mL) were labeled with 4 *μ*g/mL calcein‐AM for 45 min at 37°C. Cells were washed three times with HEPES buffer, resuspended at a concentration of 4 × 10^6^/mL and 0.5 mL was added to confluent mouse cardiac myocyte cell layer. At the end of the incubation time, nonadherent cells were removed and adherent cells and cardiac myocytes were lysed in 1 mL of 0.2% Triton X‐100. Calcein fluorescence in each sample was measured at an excitation wavelength of 485 nm and an emission wavelength of 530 nm. The percent cell adherence in each sample was calculated based upon the fluorescence measured in 0.5 mL of RAW 264.7 cell suspension.

### Polymorphonuclear leukocyte (PMN) adherence

Blood (80 mL) was obtained from healthy adults and layered over an equal volume of Polymorphprep (AxisShield, Oslo, Norway) in 50‐mL conical tubes. Tubes were spun at 500 g for 30 min at 20°C. The buffy coat at the sample–medium interface consisting of PMN was removed, washed, and resuspended in 5 mL of ice‐cold Hank's balanced salt solution (HBSS), and cells were counted. HCAEC grown on a 12‐mm plate were washed twice with HBSS. After appropriate pretreatment of endothelial cells or PMN, 0.5 mL of PMN suspension (4 × 10^6^ cells/mL) in HBSS was added to each of the wells and incubated for 10 min at room temperature. Medium and unbound cells were removed and discarded. Plates were washed twice with prewarmed Dulbecco's phosphate‐buffered saline (DPBS). Adherent PMN and endothelial cells were lysed in 1 mL of 0.2% Triton X‐100. For maximal binding, a 0.5‐mL aliquot of PMN suspension plus 0.5 mL of 0.2% Triton X‐100 was used. To measure myeloperoxidase activity, we added 400 *μ*L of cell lysate to 1 mL of phosphate‐buffered saline (PBS), 1.2 mL of Hanks’ buffer‐bovine serum albumin (BSA), and 0.2 mL of 3,3‐dimethoxybenzidine, and 0.2 mL of 0.05% H_2_O_2_ was added. After 15 min, 0.2 mL of 1% NaN_3_ was added to stop the reaction, and absorbance was measured using a 4050 ultraviolet/Visible spectrophotometer (Biochrom, Cambridge, UK) at 460 nm.

### Surface expression of adhesion molecules

HCAEC were grown to confluence in 16‐mm culture dishes and were incubated with *T. cruzi* (MOI 0.2) for up to 96 h. At the end of the incubation, cells were fixed with 1% paraformaldehyde and incubated overnight at 4°C. Cells were then washed three times with PBS and then blocked with Tris‐buffered saline‐Tween supplemented with 0.8% BSA (wt/vol) and 0.5% fish gelatin (wt/vol) for 1 h at 24°C. Appropriate primary antibody (1:50; Santa Cruz Biotechnology, Santa Cruz, CA) was used before treatment with horseradish peroxidase‐conjugated rabbit anti‐goat secondary antibody (1:5,000; Santa Cruz Biotechnology, Santa Cruz, CA). Subsequently, each well was incubated in the dark with the 3,3′,5,5′‐tetramethylbenzidine liquid substrate system and color development measured at 450 nm.

### Statistical analysis

Statistical comparison of values was performed by student's *t‐*test or one‐way analysis of variance with post hoc analysis performed using Dunnet's test. All results are expressed as mean ± SEM. Statistical significance was considered to be *P *<**0.05.

## Results

### *Trypanosoma cruzi* infection of HCAEC

**Figure 1.**
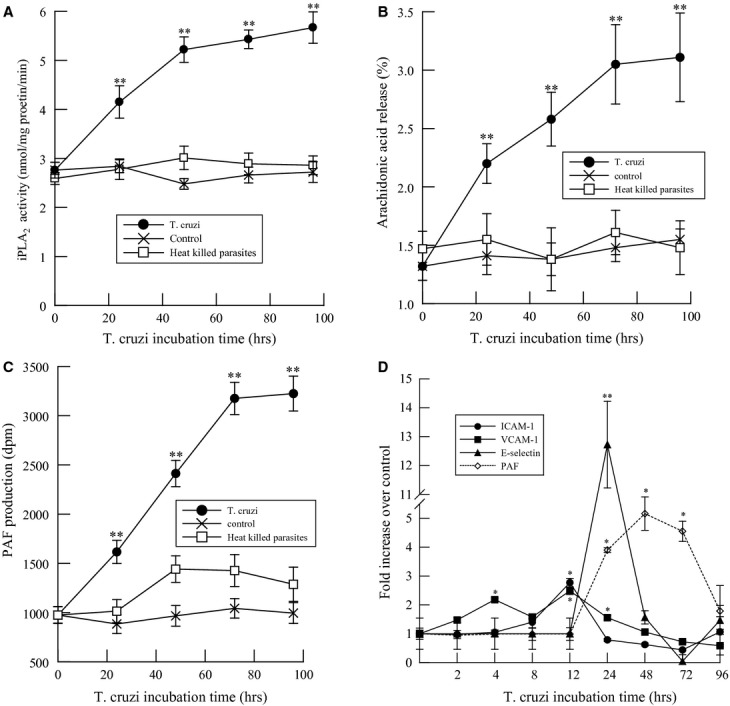
Changes in calcium‐independent phospholipase A_2_ (iPLA_2_) activity (A), arachidonic acid release (B), platelet‐activating factor (PAF) production (C), and cell surface expression of ICAM‐1, VCAM‐1, and E‐selectin (D) in human coronary artery endothelial cells infected with *Trypanosoma cruzi* (MOI 0.2, untreated ● or heat‐killed □ in A–C) for up to 96 h. Values shown are means ± SEM for four separate cell cultures. *P < 0.05, **P < 0.01 when compared to uninfected controls.

HCAEC were incubated with *T. cruzi* for up to 96 h and iPLA_2_ activity, arachidonic acid release, PAF production, and expression of cell surface adhesion molecules were measured (Fig. 1). *Trypanosoma cruzi* (MOI 0.2) infection of HCAEC increased PLA_2_ activity measured in the presence of 10 mmol/L EGTA (iPLA_2_ activity) that was significant after 24 h of infection and remained increased over 96 h (Fig. 1A). Accompanying the increase in iPLA_2_ activity, we measured a significant increase in arachidonic acid release (Fig. 1B) and PAF production (Fig. 1C). When HCAEC were incubated with heat‐killed *T. cruzi*, no significant changes in iPLA_2_ activity, arachidonic acid release, or PAF production were observed (Fig. 1A–C).

Adhesion molecules expressed on the surface of endothelial cells could significantly contribute to inflammatory cell adherence and transmigration, and may work synergistically with PAF. After infection of HCAEC with *T. cruzi*, we observed increased cell surface expression of intercellular adhesion molecule‐1 (ICAM‐1), vascular cell adhesion molecule‐1 (VCAM‐1), and E‐selectin (Fig. 1C) prior to the observed increase in PAF production (Fig. 1C). As adhesion molecules are upregulated immediately after infection, they could contribute to the initial tethering of inflammatory cells to the endothelium prior to attachment and activation via the PAF–PAF receptor interaction. The involvement of multiple molecules highlights the importance of the recruitment of circulating leukocytes, and this redundancy may allow for compensation in the absence of any single adhesion molecule.

**Figure 2.**
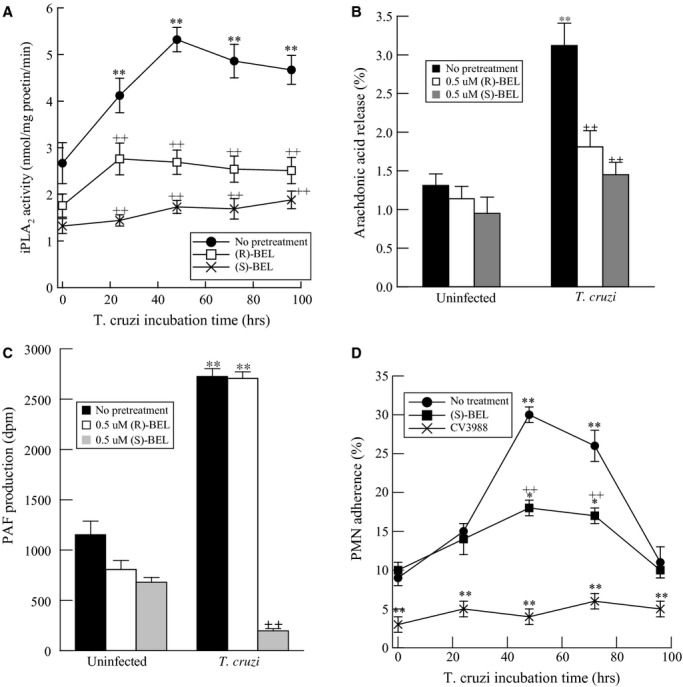
Calcium‐independent phospholipase A_2_ (iPLA_2_) activity (A), arachidonic acid release (B), platelet‐activating factor (PAF) production (C), and adherence of polymorphonuclear leukocytes (PMN) (D) in human coronary artery endothelial cells infected with *Trypanosoma cruzi* (MOI 0.2) for up to 96 h. Cells were pretreated with 0.5 *μ*mol/L (*R*)‐bromoenol lactone (BEL) or (*S*)‐BEL (10 min) prior to infection with *T. cruzi*. Values shown are means ± SEM for four separate cell cultures. **P < 0.01 when compared with uninfected controls. ^++^*P* < 0.01 when comparing values in the presence or absence of BEL.

To determine which iPLA_2_ isoform was involved in *T. cruzi*‐mediated arachidonic acid and PAF production, we pretreated HCAEC with (*R*)‐ or (*S*)‐BEL to inhibit iPLA_2_*γ* or iPLA_2_*β* selectively (Jenkins et al. 2002) prior to infection with *T. cruzi*. Treatment with (*R*)‐ or (*S*)‐BEL resulted in a significant reduction in basal iPLA_2_ activity and inhibition of iPLA_2_ activation in response to *T. cruzi* infection (Fig. 2A). Inhibition of iPLA_2_*β* activity with (*S*)‐BEL was significantly greater than corresponding concentrations of (*R*)‐BEL used to inhibit iPLA_2_*γ* (Fig. 2A), suggesting that iPLA_2_*β* activity is predominant in HCAEC.

We measured arachidonic acid release and PAF production in the presence or absence of (*R*)‐ or (*S*)‐BEL at 48 h post infection with *T. cruzi*. Endothelial cells showed a significant increase in arachidonic acid release (Fig. 2B) and PAF production (Fig. 2B) in response to *T. cruzi* infection. Although arachidonic acid release was inhibited by similar extent by (R)‐ or (S)‐BEL (Fig. 2B), PAF production was unaffected by (*R*)‐BEL pretreatment and completely inhibited by (S)‐BEL pretreatment (Fig. 2C). Hence, our data suggest that *T. cruzi*‐induced arachidonic acid release is mediated by activation of both iPLA_2_*β* and iPLA_2_*γ*, whereas increased PAF production is entirely dependent upon iPLA_2_*β* activation.

To examine the effects of iPLA_2_*β*‐mediated PAF production on inflammatory cell adherence to the endothelium, human PMNs were isolated from peripheral blood and incubated with HCAEC infected with *T. cruzi*. PMN adherence to HCAEC was assessed at 24, 48, 72, and 96 h thereafter. A time‐dependent increase in PMN adherence was observed in infected HCAEC, and maximum adherence was observed 48 h after infection with 0.2 MOI of *T. cruzi* (Fig. 2D). Pretreating HCAEC with (*S*)‐BEL prior to infection resulted in a significant reduction in neutrophil adherence, and reduced adherence was also observed when PMN were treated with the PAF receptor antagonist CV3988 (Fig. 2D). Thus, the interaction of endothelial cell PAF with the PAF receptor on inflammatory cells is critical for inflammatory cell adherence to endothelium.

**Figure 3.**
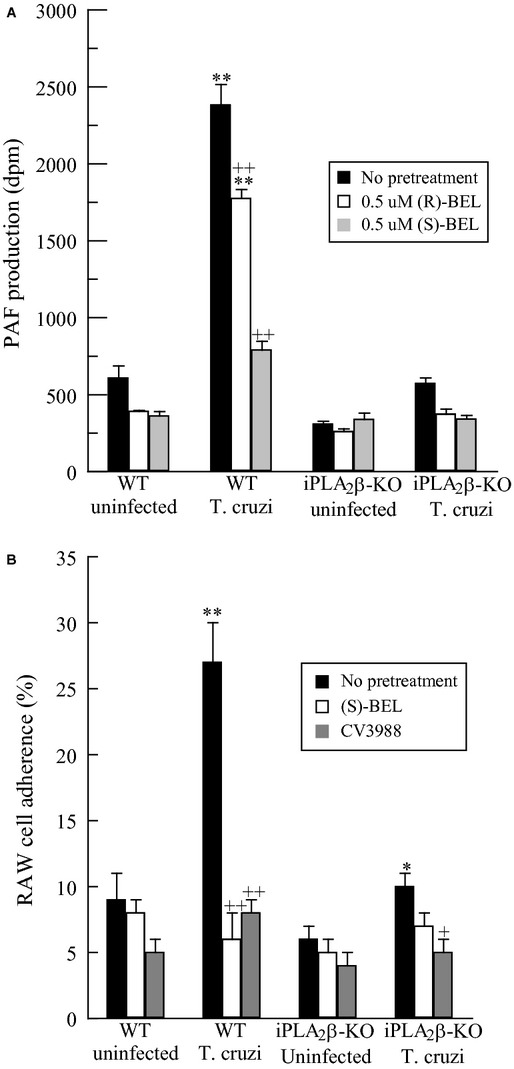
PAF production (A) and RAW 264.7 cell adherence (B) in *Trypanosoma cruzi* infected (MOI 0.2, 48 h) wild‐type or iPLA_2_*β* knockout endothelial cells following (*R*)‐ or (*S*)‐BEL pretreatment (0.5 *μ*mol/L, 10 min). *P < 0.05, **P < 0.01 when compared to uninfected cells. ^+^*P* < 0.05, ^++^*P* < 0.01 when comparing values in the presence or absence of BEL. Values shown are mean + SEM for six separate cell cultures.

To further demonstrate involvement of iPLA_2_*β* activation in increased PAF production in *T. cruzi*‐infected endothelium, cardiac endothelial cells were isolated from wild‐type and iPLA_2_*β* knockout mice. Confluent monolayers of these cells were stained for coagulation factor VIII, which is an endothelial cell‐specific marker expressed by more than 80% of the cultured cells (data not shown). After pretreatment with either (*R*)‐ or (*S*)‐BEL, endothelial cells were infected with *T. cruzi* (MOI 0.2) for 48 h and PAF production was measured. Infection with *T. cruzi* resulted in a significant increase in PAF production by wild‐type cardiac endothelial cells (Fig. 3A). Pretreatment of wild‐type endothelial cells with (*S*)‐BEL significantly inhibited the *T. cruzi*‐induced production of PAF, whereas pretreatment with (*R*)‐BEL had much less of an effect (Fig. 3A). No increase in PAF was observed when iPLA_2_*β*‐KO cardiac endothelial cells were infected with *T. cruzi*. These data suggest that iPLA_2_*β* is required for mouse cardiac endothelial cell PAF production induced by infection with *T. cruzi*. Adherence of RAW 264.7 murine macrophage/monocyte cells to cardiac endothelial cells isolated from wild‐type and iPLA_2_*β*‐KO mice following infection with *T. cruzi* was determined (Fig. 3B). After 48 h, infected wild‐type endothelial cells exhibited a significant increase in RAW 264.7 cell adherence compared to uninfected cells (Fig. 3B). Pretreatment with the iPLA_2_*β* inhibitor (*S*)‐BEL resulted in a marked reduction in RAW 264.7 cell adherence to *T. cruzi*‐infected wild type cardiac endothelial cells (Fig. 3B). No increased adherence of RAW 264.7 cells to iPLA_2_*β*‐KO cardiac endothelial cells was observed after infection with *T. cruzi* in the presence or absence of (*S*)‐BEL (Fig. 3B). Pretreatment of RAW 264.7 cells with the PAF receptor antagonist CV3988 prior to addition to *T. cruzi*‐infected endothelial cells resulted in complete inhibition of RAW 264.7 cell adherence to the cardiac endothelial cells under all experimental conditions (Fig. 3B). Thus, the increase in RAW 264.7 cell adherence to *T. cruzi*‐infected endothelial cells requires iPLA_2_*β*‐mediated PAF production.

## Discussion

Previous studies have indicated that iPLA_2_*β* is responsible for the majority of cardiac endothelial cell iPLA_2_ activity (Sharma et al. 2011). In previous studies, activation of endothelial cell iPLA_2_*β* by the proteases thrombin or tryptase results in PAF production (Sharma et al. 2011). PAF is considered an important mediator in regulating the transmigration of inflammatory cells across the endothelium and in facilitating their recruitment to areas of injury or infection (Prescott et al. 2002). Endothelial cell surface PAF interacts with the PAF receptor on the surface of inflammatory cells, and this results in activation, adhesion, and migration of leukocytes. Activated leukocytes generate reactive oxygen species, cytokines, and degradative enzymes at the site of injury in an attempt to restrict and eliminate the inflammatory stimulus, but these mediators can cause excessive inflammation that leads to tissue damage. Thus, these processes must be carefully regulated.

Our studies were designed to determine the role of endothelial cell iPLA_2_*β* during acute *T. cruzi* infection. Studies with HCAEC demonstrate that there is increased endothelial cell PAF production after infection with *T. cruzi* that requires iPLA_2_*β* (Fig. 2A). A corresponding increase in PMN adherence to the endothelium is also observed in this context (Fig. 2D). Pharmacological inhibition of iPLA_2_*β* with (*S*)‐BEL resulted in inhibition of PMN adherence to *T. cruzi*‐infected HCAEC (Fig. 2D). Additionally, blockade of the PAF‐R with the antagonist CV3988 on the PMN surface resulted in decreased adherence of neutrophils to the endothelium in both uninfected and *T. cruzi*‐infected HCAEC (Fig. 2D). These results highlight the importance of the interaction between endothelial cell PAF and PAF‐R on the inflammatory cell surface for adherence of inflammatory cells to the endothelium and hence their subsequent recruitment to the appropriate site.

Infecting HCAEC with *T. cruzi* resulted in an increase in cell surface expression of adhesion molecules as early as 4 h post infection, with a later increase in PAF production at 24 h. However, PMN adherence was not significantly increased until 48 h post infection. The time course of PMN adherence correlated with that for PAF accumulation rather than upregulation of adhesion molecules. There are multiple mediators involved in leukocyte adhesion and transmigration across the endothelium, and such biological redundancy underscores the importance of this process. Thus, even though our data indicate that PAF appears to be critical for this process in vitro, it is possible that its absence alone may not significantly affect cardiac inflammation in vivo. A number of reported observations indicate that adhesion molecules expressed on the endothelial cell surface are also important in leukocyte recruitment. Tanowitz et al. have demonstrated that infection of human umbilical vein endothelial cells (HUVECs) with *T. cruzi* results in NF‐*κ*B activation and that this leads to an increased expression of adhesion molecules (Huang et al. 1999). Michailowsky et al. (2004) have demonstrated that interferon γ (IFN*γ*) induces expression of adhesion molecules by vascular endothelium and that ICAM‐1 plays a critical role in leukocyte migration in acute *T. cruzi* infection.

Chronic Chagas’ disease is associated with cardiomyopathy involving impaired endothelial cell function that can contribute to microvascular abnormalities such as focal ischemia or microthrombus formation. Recently, Molina‐Berrios et al. (2013) demonstrated the presence of endothelial cell dysfunction markers, including soluble forms of ICAM and E‐selectin in the plasma of *T. cruzi*‐infected mice after 90 days of infection. Prolonged endothelial cell activation in chronic Chagas’ disease may also be associated with increased PAF production as a result of iPLA_2_ activation.

Studies using endothelial cells isolated from hearts of wild‐type and iPLA_2_*β* knockout mice also indicated that iPLA_2_*β* is necessary for *T. cruzi*‐induced PAF production (Fig. 3A). Wild‐type cardiac endothelial cells produced greater amounts of PAF after infection with *T. cruzi*, and this was prevented by pretreatment with the iPLA_2_*β* inhibitor (*S*)‐BEL. Endothelial cells isolated from iPLA_2_*β*‐knockout mice failed to increase PAF production after *T. cruzi* infection. Similarly, mouse RAW 264.7 macrophage‐like cells also exhibited increased adherence to mouse cardiac endothelial cells with the rise in iPLA_2_*β*‐mediated PAF production that occurred after *T. cruzi* infection (Fig. 3B). Taken together, these studies suggest that iPLA_2_*β* deficiency results in reduced endothelial cell PAF production and that this might impair recruitment of inflammatory cells to cardiac tissue in the acute and chronic phases of Chagas’ disease. Although our data indicate that the absence of iPLA_2_*β* results in impaired PMN adherence via reduced endothelial cell PAF production, iPLA_2_‐mediated hydrolysis of membrane phospholipids results in the direct or indirect production of several inflammatory metabolites (Six and Dennis 2000; Burke and Dennis 2009; Dennis et al. 2011). Thus, we cannot rule out that other iPLA_2_*β*‐catalyzed metabolites are not involved in PMN adherence. We have demonstrated previously that the absence of iPLA_2_*β* does not inhibit coronary artery endothelial cell eicosanoid generation completely (Sharma et al. 2011), but we have shown that lysoplasmenylcholine increases PMN adherence and cell surface expression of adhesion molecules (White et al. 2007). Thus, it is likely that other downstream metabolites of iPLA_2_*β*‐catalyzed membrane phospholipid hydrolysis may play a role in inflammatory cell adherence to the endothelium.

In conclusion, our data demonstrate that *T. cruzi* infection of endothelial cells results in iPLA_2_*β* activation and increased PAF production. Pretreatment of endothelial cells with an iPLA_2_*β* inhibitor or inflammatory cells with a PAF receptor antagonist demonstrate that blocking the PAF–PAF receptor interaction is sufficient to inhibit adherence of inflammatory cells to the endothelium in vitro; however, further studies are warranted to determine whether the absence of endothelial cell iPLA_2_*β* results in impaired inflammatory cell recruitment in vivo.

## Conflict of Interest

None declared.
